# Study of cell differentiation by phylogenetic analysis using histone modification data

**DOI:** 10.1186/1471-2105-15-269

**Published:** 2014-08-08

**Authors:** Nishanth Ulhas Nair, Yu Lin, Ana Manasovska, Jelena Antic, Paulina Grnarova, Avinash Das Sahu, Philipp Bucher, Bernard ME Moret

**Affiliations:** School of Computer and Communication Sciences, École Polytechnique Fédérale de Lausanne (EPFL), EPFL IC IIF LCBB, INJ 211 (Batiment INJ), Station 14, CH-1015 Lausanne, Switzerland; Department of Computer Science and Engineering, University of California, San Diego, San Diego USA; Department of Computer Science, University of Maryland, College Park, Maryland USA; School of Life Sciences, École Polytechnique Fédérale de Lausanne (EPFL), Lausanne, Switzerland; Swiss Institute of Bioinformatics, Lausanne, Switzerland

**Keywords:** Cell differentiation, Development, Cell-type trees, Epigenomics, Histone modifications, Phylogenetics, Evolution of cell types

## Abstract

**Background:**

In cell differentiation, a cell of a less specialized type becomes one of a more specialized type, even though all cells have the same genome. Transcription factors and epigenetic marks like histone modifications can play a significant role in the differentiation process.

**Results:**

In this paper, we present a simple analysis of cell types and differentiation paths using phylogenetic inference based on ChIP-Seq histone modification data. We precisely defined the notion of cell-type trees and provided a procedure of building such trees. We propose new data representation techniques and distance measures for ChIP-Seq data and use these together with standard phylogenetic inference methods to build biologically meaningful cell-type trees that indicate how diverse types of cells are related. We demonstrate our approach on various kinds of histone modifications for various cell types, also using the datasets to explore various issues surrounding replicate data, variability between cells of the same type, and robustness. We use the results to get some interesting biological findings like important patterns of histone modification changes during cell differentiation process.

**Conclusions:**

We introduced and studied the novel problem of inferring cell type trees from histone modification data. The promising results we obtain point the way to a new approach to the study of cell differentiation. We also discuss how cell-type trees can be used to study the evolution of cell types.

**Electronic supplementary material:**

The online version of this article (doi:10.1186/1471-2105-15-269) contains supplementary material, which is available to authorized users.

## Background

In developmental biology, the process by which a less specialized cell becomes a more specialized cell type is called cell differentiation. Since all cells in one individual organism have the same genome, epigenetic factors and transcriptional factors play an important role in cell differentiation [1–3]. Thus a study of epigenetic changes among different cell types is necessary to understand cell development.

Histone modifications form one important class of epigenetic marks; such modifications have been found to vary across various cell types and to play a role in gene regulation [4]. Histones are proteins that package DNA into structural units called nucleosomes [5]. These histones are subject to various types of modifications (methylation, acetylation, phosphorylation, and ubiquitination), modifications that alter their interaction with DNA and nuclear proteins. In turn, changes in these interactions influence gene transcription and genomic function. In the last several years a high-throughput, low-cost, sequencing technology called ChIP-Seq has been used in capturing these histone marks on a genome-wide scale [6, 7]. A study of how histone marks change across various cell types could play an important role in our understanding of developmental biology and how cell differentiation occurs, particularly as the epigenetic state of chromatin is inheritable across cell generations [8].

In this paper, we provide a definition for a *cell-type tree*. Cell-type trees are trees which represent the relationships between various cell-types. The nodes of this tree represent cell-types while the edges between two nodes tell us that one cell-type is differentiated from some cells of the other cell-type. It is not necessary that these various cell-types come from one individual, and therefore cell-type trees are different from cell-lineage trees. Cell-lineage trees, reconstructed from genomic variability caused by somatic mutations, represent the history of cell division in one individual organism from the very first cell, the zygote [9]. However we know that almost the entire genome (within one individual) is the same across cell-types; and it is also highly similar between individuals of the same species. However we know that epigenomic states are different across various cell types. So it is possible that in the cell differentiation process, a complex interplay between histone modifications, DNA methylation, transcription factors etc. plays an important role in how cells of various cell-types in one organism behave differently although the genome is almost same. Therefore in this study we attempt to build cell-type trees by looking at histone modification data. Currently we look at only some histone modifications for the sake of simplicity. We do this to see if there is a link between histone modifications and cell differentiation. We note that in literature certain clustering techniques like hierarchical clustering have been used to cluster cell types using various kinds of data. For example, in [10] unsupervised hierarchical clustering of whole genome expression data was done for some cell-types.

Since cell differentiation transforms less specialized cell types into more specialized ones and since most specialized cells of one organ cannot be converted into specialized cells of some other organ, the paths of differentiation together form a tree, in many ways similar to the phylogenetic trees used to represent evolutionary histories. In evolution, present-day species have evolved from some ancestral species, while in cell development the more specialized cells have evolved from less specialized cells. Moreover, observed changes in the epigenetic state are inheritable, again much as mutations in the genome are (although, of course, through very different mechanisms and at very different scales); and in further similarity, epigenetic traits are subject to stochastic changes, much as in genetic mutations. (It should be noted that we are interested here in populations of cells of a certain type, not all coming from the same individual, rather than in developmental lineages of cells within one individual). Finally, one may object that derived and more basic cell types coexist within the body, while phylogenetic analysis places all modern data at the leaves of the tree and typically qualifies internal nodes as “ancestral”. However, species in a phylogenetic tree correspond to paths, not to nodes. In particular, a species that has survived millions of years until today and yet has given rise to daughter species, much like a basic cell type that is observed within the organism, but from which derived cell types have also been produced and observed, is simply a path to a leaf in the tree, a path along which changes are slight enough not to cause a change in identification. (The time scale makes such occurrences unlikely in the case of species phylogenies, but the framework is general enough to include them).

Therefore it may be possible to use or adapt some of the techniques used in building phylogenetic trees for building *cell-type trees*. We defined the concept of cell-type trees in a previous work [11]. The major difference between phylogenetic trees and cell-type trees is that functional changes in cell differentiation are primarily driven by programmed mutational events rather than by selection. An immediate consequence is that the design of an “evolutionary” model has hardly begun in sharp contrast to sequence evolution. However, note that the program of mutational events is itself the result of evolution, so that, as observed by Arendt [12], the cell differentiation tree often recapitulates the phylogeny of cell types. Thus we felt justified to apply phylogenetic methods to the analysis of cell types.

In this paper, we provide evidence that such a scenario is possible. We do this by proposing new data representation techniques, distance measures, then by applying standard phylogenetic methods to produce biologically meaningful results. We used data on a few histone modifications (but mostly on H3K4me3) for many cell types, including replicate data, to construct cell-type trees—to our knowledge, these are the first such trees produced by computational methods. We show that preprocessing the data is very important: not only are ChIP-Seq data fairly noisy, but the ENCODE data are based on several individuals and this adds an independent source of noise. We show how various patterns of histone modification change during the cell-differentiation process and the biological significance of it. We also outline some of the computational challenges in the analysis of cell differentiation, opening new perspectives that may prove of interest to computer scientists, biologists, and bioinformaticians. We also discuss how these cell-type trees can be used to study the evolution of cell types.

## Results and discussion

### Model of differentiation for histone marks

We assume that histone marks can be independently gained or lost in regions of the genome as cells differentiate from a less specialized type to a more specialized one. Histones marks are known to disappear from less specialized cell types or to appear in more specialized ones and are often correlated with gene expression, so our assumption is reasonable. The independence assumption simply reflects our lack of knowledge, but it also enormously simplifies computations.

### Data representation techniques

The analysis of ChIP-Seq data typically starts with a peak-finding step that defines a set of chromosomal regions enriched in the target molecule. We therefore use peak lists as the raw data for our study. We use both publically available peak lists (give in ENCODE database) and also define our own ‘peak-finder’ which basically identifies regions of the genome which have significant amounts of histone modification signal (see sub-section “Peak-finding” described later). We can decide on the presence or absence of peaks at any given position and treat this as a binary character, matching our model of gain or loss of histone marks. Since all of the cell types have the same genome (subject only to individual SNPs or varying copy numbers), we can compare specific regions across cell types. Therefore we code the data into a matrix in which each row is associated with a different ChIP-Seq library (a different cell type or replicate), while each column is associated with a specific genomic region.

We use two different data representations for the peak data for each cell type. Our first method is a simple windowing (or binning) method. We divide the genome into bins of certain sizes; if the bin contains at least one peak, we code it 1, otherwise we code it 0. The coding of each library is thus independent of that of any other library.

Our second method uses overlap and takes into account all libraries at once. We first find interesting regions in the genome, based on peaks. Denote the *i*th peak in library *n* as , where  and  are the left and right endpoints (as basepair indices). Consider each peak as an interval on the genome (or on the real line) and build the *interval graph* defined by all peaks in all libraries. An interval graph has one vertex for each interval and an edge between two vertices whenever the two corresponding intervals overlap [13]. We simply want the connected components of the interval graph.

#### Definition 1

An interval in the genome is an *interesting region* iff it corresponds to a connected component of the interval graph.

A straight forward algorithm to identify these interesting regions in linear time is shown in the Methods section. For a given collection of libraries, these interesting regions have a unique representation. We assume that it is in these interesting regions that histone marks are lost or gained and we consider that the sizes of the peak regions (which depends at least in part on the experimental procedures and is typically noisy) does not matter. Our major reason for this choice of representation is noise elimination: since the positioning of peaks and the signal strength both vary from cell to cell as well as from test to test, we gain significant robustness (at the expense of detail) by merging all overlapping peaks into one signal, which we use to decide on the value of a single bit. The loss of information may be illusory (because of the noise), but in any case we do not need a lot of information to build a phylogeny on a few dozen cell types.

### Phylogenetic analysis

Phylogenetic analysis attempts to infer the evolutionary relationships of modern species or *taxa*—they could also be proteins, binding sites, regulatory networks, etc. The best tools for phylogenetic inference, based on maximum parsimony (MP) or maximum likelihood (ML), use established models of sequence evolution, something for which we have no equivalent in the context of cell differentiation. However, one class of phylogenetic inference methods, so-called distance-based methods, are founded on hierarchical clustering under some suitable measure of pairwise distance for similarity. This type of method is directly applicable to our problem, provided we can define a reasonable measure of distance, or similarity between cell types in terms of our data representations. (We are not implying that models of differentiation do not exist nor that they could not be derived, but simply stating that none exist at present that could plausibly be used for maximum-likelihood phylogenetic inference). Finally note that, with 0/1 data, we can also use an MP method, in effect assuming that all changes are equally likely.

In a cell type tree, most cell types coexist in the present; thus at least some of them can be found both at leaves and at internal nodes. (We may not have data for all internal nodes, as we cannot claim to have observed all cell types). Fortunately, phylogenetic inference still works in such cases: as mentioned earlier, when the same taxon should be associated with both a leaf and an internal node, we should simply observe that each edge on the path from that internal node to that leaf is extremely short, since that distance between the two nodes should be zero (within noise limits). The tree inferred will have the correct shape; however, should we desire to reconstruct the basic cell types, then we would have to *lift* some of the leaf data by copying them to some internal nodes.

Of the many distance-based methods, we chose the most commonly used one, Neighbor-Joining (NJ) [14]. While faster and possibly better distance-based methods exist, such as FastME [15], it was not clear that their advantages would still obtain in this new domain; and, while very simple, the NJ method has the advantage of not assuming a constant rate of evolution across lineages. In each of the two data representation approaches, we compute pairwise distance between two libraries as the Hamming distance of their representations. (The Hamming distance between two strings of equal length is the number of positions at which corresponding symbols differ). We thus obtain a distance matrix between all pairs of histone modification libraries; running NJ on this matrix yields an unrooted tree. For MP, we used the TNT software [16].

### On the inference of ancestral nodes

We mentioned that lifting some of the leaf data into internal nodes is the natural next step after tree inference. However, in general, not all internal nodes can be labelled in this way, due mostly to sampling issues: we may not have observed the type that should be associated with a particular internal node, or we may be missing enough fully differentiated types that some internal tree nodes do not correspond to any real cell type. Thus we are faced with a problem of ancestral reconstruction and, more specifically, with three distinct questions:

- For a given internal node, is there a natural lifting from a leaf?
- If there is no suitable lifting, is the node nevertheless a natural ancestor—i.e., does it correspond to a valid (real) cell type?
- If the node has no suitable lifting and does correspond to a valid cell type, can we infer its data representation?

These are hard questions, in terms of both modelling and computational complexity; they are further complicated by the noisy nature of the data. Such questions remain poorly solved in standard phylogenetic analysis; in the case of cell-type trees, we judged it best not to address these problems until the tree inference part is better understood and more data are analyzed.

### Peak-finding

Since our algorithms work on peak data, one needs to use some peak finder to convert the ChIP-Seq histone modification libraries into peaks. One can use any peak finder. We used the publically available peaks given by the ENCODE project for our analysis.

Since we found the peaks to be noisy, we also used the MACS2 peak finder (version number 2.0.10.20131216), which is a newer version of the popular MACS peak finder [17]. MACS2 was run using the input control data with its default parameters.

### Experimental design

The histone modification ChIP-Seq data were taken from the ENCODE project database (UW ENCODE group) for human (hg19) data [18]. We carried out experiments on H3K4me3 and H3K27me3 histone mark data from University of Washington (UW) ENCODE group and on H3K4me1, H3K9me3, H3K27ac histone mark data from Broad ENCODE group [18]. H3K4me3 is a well studied histone mark usually associated with gene activation, while the less well studied H3K27me3 is usually associated with gene repression [19]. We used data for cell types classified as “normal” and for embryonic stem cells—we did not retain cancerous or EBV cells as their differentiation processes might be completely distinct from those of normal cells. The ENCODE project provides peaks of ChIP-Seq data for each replicate of each cell type. We therefore used their peaks as the raw input data for our work. For the windowing representation, we used bins of 200 bp: this is a good size for histone marks, because 147 bp of DNA wrap around the histone and linker DNA of about 80 bp connect two histones, so that each bin represents approximately the absence or presence of just one histone modification. We programmed our procedures in *R* and used the NJ implementation from the *ape* library in *R*.

Table 1 shows the list of the 37 cell types (72 libraries including all replicates) used for H3K4me3 data and 13 cell types (23 libraries including all replicates) for H3K27me3 data, giving for each an abbreviation and a short description. The table also shows the 10, 11, and 12 cell types used for H3K4me1, H3K9me3, H3K27ac respectively. In addition, the cells are classified into various groups whose names are based on their cell type. Keratinocytes (NHEK) is included in the Epithelial group. We have two replicates for most cell types, but only one replicate for types HCFaa, HFF, and CD14, and three replicates for CD20. (CD20(1) is a B-cell from an African-American individual while CD20(2) and CD20(3) are from a Caucasian individual). The replicates are biological replicates, i.e., the data come from two independent samples. For human Embryonic Stem Cells (hESC) we have data for different days of the cell culture (day 0, 2, 5, 9, 14) for H3K4me3 and H3K27me3 data, so we shall use hESC D2 (or hESC T2) to mean data for hESC cells on day 2. For each cell type, we shall mention the replicate number in brackets, unless the cell type has only one replicate. All our experiments are done using the neighbor-joining distance based approach unless otherwise mentioned. More information about where we collected ENCODE peak data from is given in the Additional file 1.

**Table 1:** **Cell types, short description, and general group for H3K4me3, H3K27me3, H3K4me1, H3K9me3, H3K27ac data**

Cell name	Short description	Group	H3K4me3	H3K27me3	H3K4me1	H3K9me3	H3K27ac
AG04449	Fetal buttock/thigh fibroblast	Fibroblast	*✓*				
AG04450	Fetal lung fibroblast	Fibroblast	*✓*	*✓*			
AG09319	Gum tissue fibroblasts	Fibroblast	*✓*				
AoAF	Aortic adventitial fibroblast cells	Fibroblast	*✓*				
BJ	Skin fibroblast	Fibroblast	*✓*	*✓*			
CD14	Monocytes-CD14+ from human leukapheresis production	Blood	*✓*	*✓*	*✓*	*✓*	*✓*
CD20(1)	B cells replicate, African American	Blood	*✓*				*✓*
CD20(2) and CD20(3)	B cells replicates, Caucasian	Blood	*✓*				
hESC	Undifferentiated embryonic stem cells	hESC	*✓*	*✓*	*✓*	*✓*	*✓*
HAc	Astrocytes-cerebellar	Astrocytes	*✓*				
HAsp	Astrocytes spinal cord	Astrocytes	*✓*				
HBMEC	Brain microvascular endothelial cells	Endothelial	*✓*				
HCFaa	Cardiac fibroblasts- adult atrial	Fibroblast	*✓*				
HCF	Cardiac fibroblasts	Fibroblast	*✓*				
HCM	Cardiac myocytes	Myocytes	*✓*				
HCPEpiC	Choroid plexus epithelial cells	Epithelial	*✓*				
HEEpiC	Esophageal epithelial cells	Epithelial	*✓*				
HFF	Foreskin fibroblast	Fibroblast	*✓*				
HFF MyC	Foreskin fibroblast cells expressing canine cMyc	Fibroblast	*✓*				
HMEC	Mammary epithelial cells	Epithelial	*✓*	*✓*	*✓*	*✓*	*✓*
HPAF	Pulmonary artery fibroblasts	Fibroblast	*✓*				
HPF	Pulmonary fibroblasts isolated from lung tissue	Fibroblast	*✓*				
HRE	Renal epithelial cells	Epithelial	*✓*	*✓*			
HRPEpiC	Retinal pigment epithelial cells	Epithelial	*✓*				
HSMM	Skeletal muscle myoblasts	Skeletal Muscle			*✓*	*✓*	*✓*
HSMMtube	Skeletal muscle myotubes differentiated from the HSMM cell line	Skeletal Muscle			*✓*	*✓*	*✓*
HUVEC	Umbilical vein endothelial cells	Endothelial	*✓*	*✓*	*✓*	*✓*	*✓*
HVMF	Villous mesenchymal fibroblast cells	Fibroblast	*✓*				
NHA	Astrocytes (also called Astrocy)	Astrocytes			*✓*	*✓*	*✓*
NHDFAD	Adult dermal fibroblasts	Fibroblast				*✓*	*✓*
NHDF Neo	Neonatal dermal fibroblasts	Fibroblast	*✓*				
NHEK	Epidermal keratinocytes	Epithelial	*✓*	*✓*	*✓*	*✓*	*✓*
NHLF	Lung fibroblasts	Fibroblast	*✓*		*✓*	*✓*	*✓*
Osteobl	Osteoblasts (NHOst)	Osteoblasts			*✓*	*✓*	*✓*
RPTEC	Renal proximal tubule epithelial cells	Epithelial	*✓*				
SAEC	Small airway epithelial cells	Epithelial	*✓*	*✓*			
SKMC	Skeletal muscle cells	Skeletal Muscle	*✓*				
WI 38	Embryonic lung fibroblast cells	Fibroblast	*✓*				

For details see the ENCODE website [20]. The mark *✓* shows the usage of that cell type for that particular histone mark.

### H3K4me3 data on individual replicates

**Figure 1:**
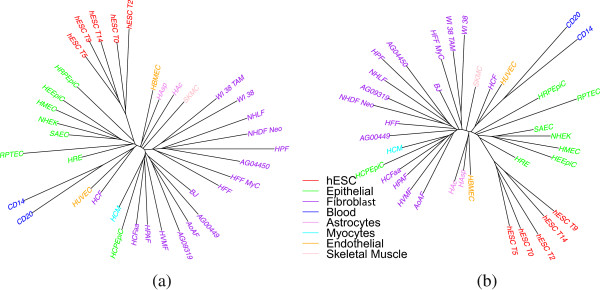
**Cell-type tree on H3K4me3 data (ENCODE peaks) using only one replicate: (a) windowing representation, (b) overlap representation.**

We report on our analyses using peak data from the ENCODE database for H3K4me3 histone modifications. We carried out the same analyses using H3K27me3 data, but results were very similar and so are not detailed here—we simply give one tree for comparison purposes. The similarity of results between the two datasets reinforces our contention that phylogenetic analyses yield biologically meaningful results on such data. We color-code trees to reflect the major groupings listed in Table 1.Figure 1 shows the trees constructed using only one replicate for each cell type using both windowing and overlap representations. The color-coding shows that embryonic stem cells and blood cells are in well separated clades of their own, while fibroblasts and epithelial cells fall in just two clades each. Even within the hESC group we see that day 0 is far off from day 14 compared to its distance from day 2. Thus epigenetic data such as histone marks do contain a lot of information about cell differentiation history.

In order to quantify the quality of the groupings, we compute the total number of cells in a subtree that belong to one group. Since our groups are based on cell type only, there could be many subdivisions possible within each group. Therefore we choose the two largest such subtrees available for each group such that each subtree contains only the leaf nodes of that group. The results are shown in Table 2: most of the cell types in each group do cluster together in the tree.

**Table 2:** **Statistics for cell-type trees on H3K4me3 data**

	hESC	Epithelial	Fibroblast	Blood	Astrocytes	Myocytes	Endothelial	Skeletal muscle	***SR***	***PD***
	(5)	(8)	(16)	(2)	(2)	(1)	(2)	(1)		(%)
WM (one replicate)-ENCODE	5,0	6,1	8,4	2,0	1,1	1,0	1,1	1,0	0.93	3.20
OM (one replicate)-ENCODE	5,0	4,1	6,3	2,0	2,0	1,0	1,1	1,0	0.92	3.94
OM (one replicate)-ENCODE-MP	5,0	4,2	6,4	2,0	1,1	1,0	1,1	1,0	0.63	-
WM (one replicate)-MACS2	5,0	4,2	14,1	2,0	2,0	1,0	1,1	1,0	0.88	5.51
OM (one replicate)-MACS2	5,0	4,2	13,3	2,0	2,0	1,0	1,1	1,0	0.89	4.84
WM (all replicates)-ENCODE	5,0	6,1	11,2	2,0	1,1	1,0	1,1	1,0	0.84	3.30
OM (all replicates)-ENCODE	5,0	4,2	9,4	2,0	2,0	1,0	1,1	1,0	0.78	3.88
WM (all replicates)-MACS2	5,0	4,2	14,1	2,0	2,0	1,0	1,1	1,0	0.63	5.31
OM (all replicates)-MACS2	5,0	4,2	15,1	2,0	2,0	1,0	1,1	1,0	0.65	5.18
WM (all replicates)-TP-ENCODE	5,0	6,1	7,4	2,0	1,1	1,0	1,1	1,0	0.81	3.73
OM (all replicates)-TP-ENCODE	5,0	4,3	8,5	2,0	2,0	1,0	1,1	1,0	0.74	3.98
OM (profile)-ENCODE	5,0	4,3	12,2	2,0	2,0	1,0	1,1	1,0	0.90	4.05

2nd to 9th columns show the number of cells (of the same type) belonging to the largest and second-largest clades; the total number of cells of that type is in the top row. Rows correspond to various methods (WM: windowing; OM: overlap; TP: top peaks with threshold of 10). The second last column shows the *SR* ratio. The last column contains the percent deviation (*PD*) of the distances between the leaves found using the NJ tree from the Hamming distance between the leaves. ENCODE means peaks from ENCODE data is used while MACS2 means peaks from MACS2 program is used. (one replicate) means only one replicate for each cell type is used, (all replicates) means all available replicates (1, 2, or 3) for each cell type is used, (profile) means a profile representation created using all replicates for each cell type is used. MP - maximum parsimony using TNT software.

Figure 1 shows long edges between (most) leaf nodes and their parents—a disquieting feature, as it casts doubt as to the robustness of the tree, parts of which could be assimilated to star-shaped trees (a tree with only one internal node and the remaining nodes being leaves). To quantify this observation, we measured the *SR* ratio, defined as , where *I* is the set of all edges connecting leaf nodes to their parents, *E* is the set of all edges in the tree, and *l*(*e*) is the length of edge *e*. If this ratio *SR* is close to 1, then the tree looks star-shaped with long branches to the leaves. This ratio was 0.93 using the windowing representation; using the overlap representation reduced it very slightly to 0.92. These long branches are due in part to the very high level of noise in the data, explaining why the overlap representation provided a slight improvement.

As a final entry in the table, we added another measure on the tree and the data. The NJ algorithm is known to return the “correct” tree when the distance matrix is ultrametric; the technical definition does not matter so much here as the consequence: if the matrix is ultrametric, then the sum of the length of the edges on the path between two leaves always equals the pairwise distance between those two leaves in the matrix. Thus one way to estimate how far the distance matrix deviates from this ideal is to compare its distances to the length of the leaf-to-leaf paths in the tree:


where *i* and *j* are leaf nodes, *NJ*(*i*,*j*) is the tree distance between *i* and *j*, and *M*(*i*,*j*) is the matrix distance between *i* and *j*. A high value of *PD* indicates that the data representations and measures do not fit well to any tree. We get very low values (of less than 4% for both windowing and overlap representations), suggesting that the distances we compute are in fact representative of a tree and thus offering confirmation of the validity of the inference.

**Figure 2:**
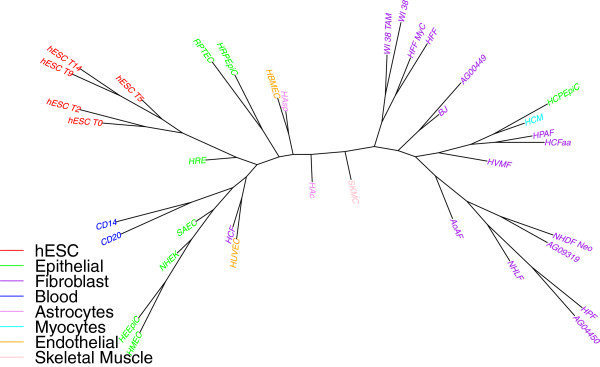
**Using maximum parsimony (TNT software) on H3K4me3 data (ENCODE peaks) using only one replicate (overlap representation).**

Finally, the trees obtained using TNT software (MP based method) are very similar but we got a better *SR* ratio as shown in Table 2. The results using TNT software for overlap representation when using only one replicate of H3K4me3 data is shown in Figure 2.

### H3K4me3 data with all replicates

**Figure 3:**
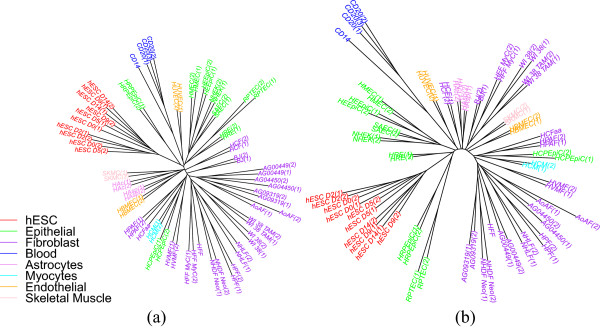
**Cell-type tree on H3K4me3 data (ENCODE peaks, using all replicates): (a) windowing representation, (b) overlap representation.**

**Figure 4:**
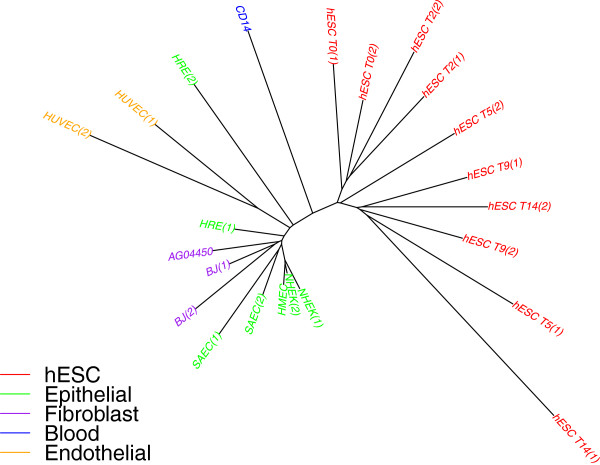
**Cell-type tree on H3K27me3 data (ENCODE peaks), using all replicates and overlap representation.**

By bringing replicates into the analysis, we can expect to see a stronger phylogenetic signal as each replicate adds to the characterization of its cell type. In particular, wherever we have two or more replicates, they should form a tight subtree of their own. We thus used our replicate data (two replicates for 33 of the 37 cell types, and three for one type, for a total of 72 libraries) in the same analysis pipeline. Figure 3 shows the differentiation trees obtained using windowing and overlap representations. We also include the same study (in overlap representation only) on H3K27me3 data in Figure 4. As expected, almost all replicates are grouped; since we usually have two replicates, we get a collection of “cherries” (pairs of leaves) where we had a single leaf before. In most cases, it is now the distance from each leaf in a cherry to their common parent that is large, indicating that the distance between the two replicates is quite large—as we can also verify from the distance matrix. This suggests much noise in the data. This noise could be at the level of raw ChIP-Seq data, but also due to the bias of peak-finding methods used—one expects a general-purpose peak finder to be biased against false negatives and more tolerant of false positives, but for our application we would be better served by the inverse bias. Another reason for the large distance is the nature of the data: these are biological replicates, grown in separate cultures, so that many random losses or gains of histone marks could happen once the cell is differentiated. Thus it may be that only a few of the variations in the data are correlated with cell differentiation. Identifying these few variations would be of high interest, but with just two replicates we are unlikely to pinpoint them with any accuracy.

Looking again at Table 2, we see that, using the windowing representation, the value of *SR* for the full set of replicates is 0.84 and that here the overlap representation, which is more effective at noise filtering, yields an *SR* value of 0.78. This is a substantial reduction and indicates that the long edges are indeed due to noise. The *PD* percentage values remain very low for both representations, so the trees we obtained do represent the data well. Note that the groupings appear (in the color-coding in the figure) somewhat better than when we used only one replicate, and the values in columns 2 through 9 of Table 2 confirm this impression.

We also include results using windowing representation on H3K4me1 data, H3K9me3 data, and H3K27ac data in Figures S8, S9, S10 respectively (see Additional file 1). We got good results on these datasets as seen from these figures as well.

### Using top peaks, masking regions, IDR analysis

In order to study the nature of the noise, we removed some of the less robust peaks. The ENCODE dataset gives a p-value for each peak listed; we kept only peaks with (negative) log p-values greater than or equal to a threshold of 10. We kept all replicates and ran the analysis again, with the results depicted in Additional file 1: Figure S1. The *PD* percentage values are again very low, so the trees once again fit the data well. The improvement looks superficially minor, but we obtained some more biologically meaningful clusters with this approach. For example, in the fibroblast group when we used only top peaks in the overlap representation, one cell type HFF moved to sub-tree containing HFF-Myc (which makes more sense as both are foreskin fibroblast cells). Such a change could be due to particularly noisy data for the HFF cells having obscured the relationship before we removed noisy peaks. Overall, removing noisy peaks further reduced the *SR* ratio from 0.78 to 0.74 for the overlap representation and from 0.84 to 0.81 for the windowing representation. To test for robustness of the method, we also ran the overlap representation on ENCODE peak data with (negative) log p-values greater than or equal to various thresholds. The results are shown in the Additional file 1: Table S1. The table shows the method works quite well in most of these thresholds.

Another typical noise-reduction procedure, much used in sequence analysis, is to remove regions that appear to carry little information or to produce confounding indications—a procedure known as masking. We devised a very simplified version of masking for our problem, for use only with replicate data, by removing any region within which at most one library gave a different result (1 instead of 0 or vice versa) from the others. In such regions, the presence of absence of peaks is perfectly conserved across all but one replicate. It is possible that replicate data differs from each other because of the noisy nature of the data or because the differences are actually present in the cells due to biological reasons. In the latter case, the differences between the two replicates are not cell type specific (as they differ among replicates), hence they are not important for our analysis. After removing such regions, we have somewhat shorter representations, but follow the same procedure. The trees returned have exactly the same topology and so are not shown; the length of edges changed very slightly, as the *SR* value decreased from 0.74 down to 0.70 using top peaks in the overlap representation.

IDR (irreproducible discovery rate) analysis [21] was carried out with a *R* script downloaded from: https://sites.google.com/site/anshulkundaje/projects/idr. We used data containing exactly 2 replicates on H3K4me3 ENCODE peak data. That is we removed CD14, CD20(1), HFF, HCFaa since they have only one replicate from the earlier used dataset. Therefore we have 34 cell types and 68 libraries (2 replicates per cell type). The IDR analysis was carried out for overlap representation at various IDR thresholds of 0.01, 0.1, 0.25 for the overlapping peaks between the two replicates for each cell type. The results are shown in Additional file 1: Table S2 and Figure S12. As shown in the table, we see a slight improvement of the clustering in epithelial cell types when using an IDR analysis. Since the IDR analysis was done on overlapping peaks, we got an *SR* ratio of 0 between two replicates due to the nature of the overlap representation.

### A better looking tree

**Figure 5:**
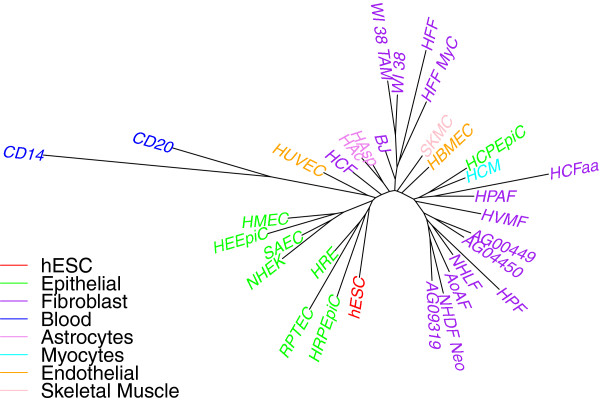
**H3K4me3 data (ENCODE peaks), overlap representation on peaks with negative log p-value ≥ 10.** Replicate leaves are removed and replaced by their parent.

Barring the addition of many replicates, the *SR* ratio of 0.70 appears difficult to reduce and yet remains high. However, the cherries of replicate pairs by themselves give an indication of the amount of “noise” (variation among individual cells as well as real noise) present in the data. We can take that noise out directly by replacing each cherry in the tree with its parent, which is a better representative of the population of this particular cell type than either of the two leaves. We carried out this removal on the tree of Additional file 1: Figure S1(b) and obtained the tree shown in Figure 5. Since hESC cells do not form clear pairs, we replaced the entire clade of hESC cells by their last common ancestor. The leaves with remaining long edges are those for which we did not have a replicate (CD14, HCFaa, and HFF).

### Using MACS2 peaks

**Figure 6:**
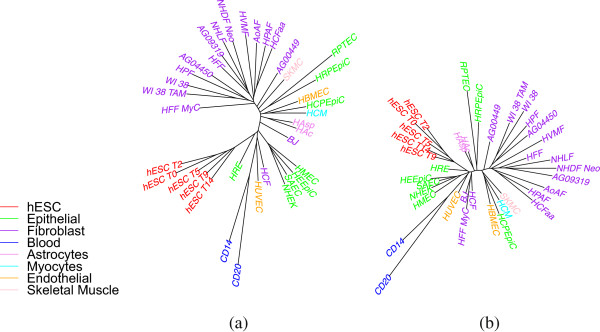
**Cell-type tree on H3K4me3 data (using one replicate) using (a) windowing representation (b) overlap representation.** Peaks generated by MACS2 method.

We look at the cell-type trees obtained using MACS2 peaks. The results are shown in Figure 6 (using only one replicate for each cell type), Additional file 1: Figure S2 (using all replicates), and Table 2. We see from the results that we get better results using MACS2 peak data than when using ENCODE peaks. This also indicates the importance of data preprocessing.

### Creating a profile using replicate data

**Figure 7:**
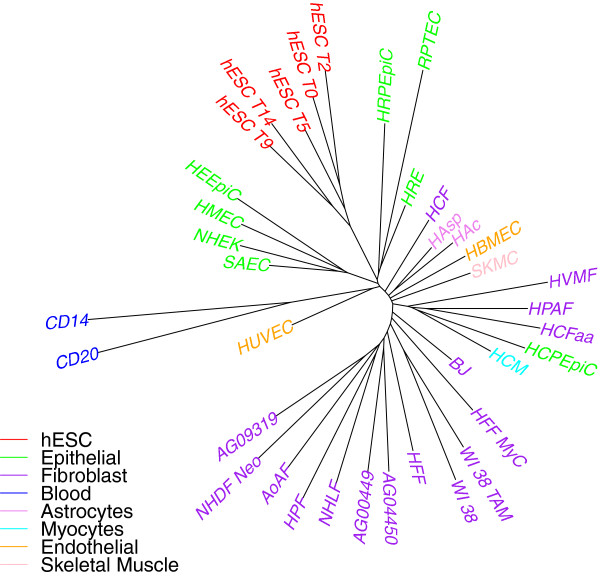
**Cell-type tree on H3K4me3 data (using profile data representation) using Overlap representation (ENCODE peaks used).**

We also show a method of creating profile of a cell type using the data representation of individual replicates. For each cell type, the profile in each bin or interesting region is represented as sum of all 1/0 (data representation value) of each replicate of that cell type in that bin or interesting region divided by the number of replicates. For example, if there are 2 replicates for one cell type, the profile at interesting region *i* would be 1 if both replicates have 1, 0 if both replicates are 0, 0.5 if one replicate is 1 and the other is 0. Using this new data representation using the profile data representation, we build trees using the neighbor-joining method. The distance between two profile representation (one for each cell type) is sum of all the absolute value of the difference between the profile values at each bin/interesting region. The results are shown in Figure 7 and Table 2. We see an improvement of results using the profile representation when compared to using all replicates or one replicate data.

### Looking at changes along specific branches of the tree

Phylogenetic analysis allows to reconstruct ancestral nodes and thus to study important branches of a tree. We are interested in the changes that happen early in development when ES cells start to differentiate into lineage-specific cell types. To this end, we selected genomic regions which are all 1s in the ES samples and 0s elsewhere allowing for one error in each group. We also selected genomic regions showing the opposite behavior. The results we show are all based on ENCODE peak lists (including replicates) using the overlap representation. We then looked at the enrichment of gene ontology (GO) and other gene annotation terms for genes adjacent to the identified genomic regions, using the GREAT website [22]. This type of analysis was carried for both H3K4me3 data and H3K27me3. The detailed results are shown in the Additional file 1.

We found 322 and 126 regions that were specifically marked by H3K4me3 in ES or non-ES cells, respectively. In the ES positive group, we found significant associations with expression in neural tissues (Additional file 1: Figure S3). This could be explained by the fact that both brain and ES cells have unusually broad expression patterns compared to other tissues. H3K4me3-depleted regions are often flanked by transcription factor genes with zing-finger domains (Additional file 1: Figure S4). The majority of these genes are probably repressed in undifferentiated ES cells.

Carrying out the same type of analysis with H3K27me3 data, we found 4036 regions that were specifically marked in ES cells, but only seven regions showing the opposite histone modification pattern. We find the ES-specific regions to be enriched near genes involved in morphogenesis, consistent with the assumption that such genes have to be repressed in undifferentiated ES cells (Additional file 1: Figure S5). By looking at the numbers of the individual classes, it appears that loss of a histone mark is a more frequent event during development than a gain of a histone mark. The imbalance is stronger for the repressive mark H3K27me3 than for the activating mark H3K4me3.

We explored the distribution of H3K4me3 and H3K27me3 modifications along various pathways of cell differentiation. As before, the analysis was done on ENCODE peak lists (including replicates) using the overlap representation. We considered only regions which do not show ambiguity between replicates. Table 3 shows the distribution of histone modification patterns over days 0, 2, 5, 9 and 14 of the ES cell differentiation time course. (Note that the all-zero pattern is not included since the overlap representation requires that a peak be found in at least one sample). We see from this table that the “all one” pattern (‘11111’) is the most dominant. We also see that patterns with one change over time such as ‘00001’, ‘00011’, ‘00111’, ‘01111’, ‘11110’, ‘11100’, ‘11000’, ‘10000’ are relatively frequent, whereas patterns involving multiple losses or gains such as ‘10101’, ‘01010’, ‘11011’ are rarely found. Patterns with a single gain followed by a loss immediately thereafter (like ‘00100’) are not so rare. However, the opposite class of patterns (like ‘11011’) is very rare. We did gene enrichment analysis on regions showing pattern ‘01000’. While analyzing H3K4me3 data, we found a great diversity of gene annotation terms, with a preponderance of terms related to proliferation and development (Additional file 1: Figure S6). While analyzing H3K27me3 data, many gene annotation terms associated with development were found—like heart development, palate development, nerve development etc. (Additional file 1: Figure S7). The gene annotation terms associated with specific histone modifications appearing on day two are compatible with a sudden response to an external stimulus activating a developmental pathway.

**Table 3:** **Analysis on paths for various days of ES cells**

Row no.	D0	D2	D5	D9	D14	H3K4me3	H3K27me3
						(total)	(total)
1	0	0	0	0	1	1075	3496
2	0	0	0	1	0	342	743
3	0	0	0	1	1	387	599
4	0	0	1	0	0	331	1459
5	0	0	1	0	1	15	97
6	0	0	1	1	0	40	112
7	0	0	1	1	1	247	461
8	0	1	0	0	0	1278	1919
9	0	1	0	0	1	14	22
10	0	1	0	1	0	9	57
11	0	1	0	1	1	30	82
12	0	1	1	0	0	60	74
13	0	1	1	0	1	5	11
14	0	1	1	1	0	9	34
15	0	1	1	1	1	147	253
16	1	0	0	0	0	450	641
17	1	0	0	0	1	11	9
18	1	0	0	1	0	11	5
19	1	0	0	1	1	14	3
20	1	0	1	0	0	24	40
21	1	0	1	0	1	6	5
22	1	0	1	1	0	10	5
23	1	0	1	1	1	101	81
24	1	1	0	0	0	630	1140
25	1	1	0	0	1	17	21
26	1	1	0	1	0	11	26
27	1	1	0	1	1	47	71
28	1	1	1	0	0	309	548
29	1	1	1	0	1	54	52
30	1	1	1	1	0	263	335
31	1	1	1	1	1	25112	10926

Table shows the number of different types of changes across various days of ES cells. Dx means day x of ES cell type. 1 and 0 represents the presence or absence of a peak as defined by the overlap representation in one region of the genome. The number of such 1-0 patterns are counted and presented in the last column.

Table 4 shows results from a similar kind of analysis along another developmental pathway comprising ES (day 0), HUVEC, and HBMEC. (These three cell types should occur one after the other during development). Table 5 shows results for yet another such developmental pathway consisting of ES (day 0), WI38, AG04550, and HPF. Again we see that the “all one” pattern is quite frequent for H3K4me3 data compared to other patterns. However such was not the case for H3K27me3. The contrasting behavior may be due to the fact that H3K4me3 is often associated with constitutive (house-keeping) genes whereas H3K27me3 primarily regulates developmental genes. From this perspective, it would be unlikely to find invariantly H3K27me3 marked regions along a complete differentiation pathway starting from ES.

**Table 4:** **Analysis on paths for ES, HUVEC, and HBMEC cell types**

H3K4me3 (row no.)	ES (D0)	HUVEC	HBMEC	Total
1	0	0	1	3407
2	0	1	0	1769
3	0	1	1	1805
4	1	0	0	5224
5	1	0	1	1415
6	1	1	0	417
7	1	1	1	23824
**H3K27me3 (row no.)**	**ES (D0)**	**HUVEC**	**HBMEC**	**Total**
1	0	1	NA	12468
2	1	0	NA	14684
3	1	1	NA	8403

Table shows the number of different types of changes for ES (day 0), HUVEC, and HBMEC cell types. 1 and 0 represents the presence or absence of a peak as defined by the overlap representation in one region of the genome. The number of such 1-0 patterns are counted and presented in the last column. NA - not applicable (because data for the cell-type is not available).

**Table 5:** **Analysis on paths for ES, WI38, AG04550, and HPF cell types**

H3K4me3 (row no.)	ES (D0)	WI38	AG04550	HPF	Total
1	0	0	0	1	2946
2	0	0	1	0	1050
3	0	0	1	1	1106
4	0	1	0	0	1670
5	0	1	0	1	382
6	0	1	1	0	879
7	0	1	1	1	4644
8	1	0	0	0	5465
9	1	0	0	1	354
10	1	0	1	0	353
11	1	0	1	1	989
12	1	1	0	0	62
13	1	1	0	1	35
14	1	1	1	0	506
15	1	1	1	1	21806
**H3K27me3 (row no.)**	**ES (D0)**	**WI38**	**AG04550**	**HPF**	**Total**
1	0	NA	1	NA	14734
2	1	NA	0	NA	20108
3	1	NA	1	NA	6342

Table shows the number of different types of changes for ES (day 0), WI38, AG04550, HPF cell types. 1 and 0 represents the presence or absence of a peak as defined by the overlap representation in one region of the genome. The number of such 1-0 patterns are counted and presented in the last column. NA - not applicable (because data for the cell-type is not available).

The bed files containing regions of the genome for which gene enrichment analysis was done are given as Additional files 2, 3, 4, 5, 6 and 7.

### Discussion on the evolutionary interpretation of cell-type trees

In this paper, we have used cell-type trees for studying cell differentiation. We used phylogenetic methods such as neighbor-joining for our work because of the similarities between cell-differentiation process and evolution (as we outlined earlier). Now we discuss how cell-type trees can be used to study the evolution of cell-types among different species.

**Figure 8:**
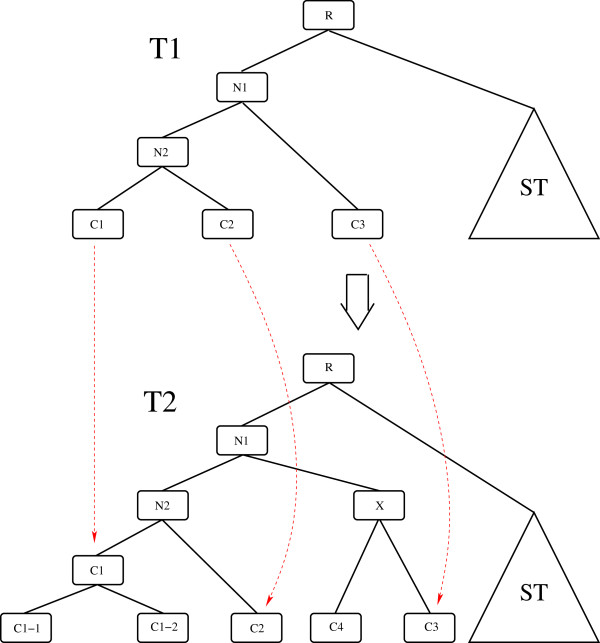
**Cell-type trees to study evolution of cell types — a constructed example is shown in this figure.** Tree *T*2 — current species *S*2. Tree *T*1 — ancestral species *S*1. *ST* — sub-tree.

Arendt [12] outlines the interrelationship between the evolution of cell types and the cell development process, mentioning that, in some cases, cell type development seems to recapitulate cell type evolution. Cell-type trees can be used to study the evolution of cell types. These trees are somewhat similar to phylogenetic trees based on gene duplication-loss models or trees build on morphology based characters. We explain the concept through an example. Figure 8 shows an constructed example of a particular current species *S*2 (bottom cell-type tree *T*2) to a particular ancestral species *S*1 (top cell-type tree *T*1). The leaf nodes in tree *T*2: *C*1−1, *C*1−2, *C*2, *C*3, *C*4 represent blood cells of the current species *S*2. The leaf nodes of tree *T*1: *C*1, *C*2, *C*3 represent blood cells of ancestral species *S*1. The internal nodes of each tree represent cell types of some ancestral species. We can see that leaf node *C*1 of tree *T*1 is the parent of leaf nodes *C*1−1 and *C*1−2 of tree *T*2. Similarly some other nodes are from one tree to another are marked by red arrows. The leaf nodes of each tree represent various blood cell types present in that species. The figure shows how the ancestral nodes in *S*2 could be leaf nodes in ancestor *S*1. Other possibilities are also shown. One possibility is that current species have more blood cell types than an ancestral species and this is captured by a cell-type tree. Thus the cell-type trees we generate using histone modification data could also be used to study the evolution of cell types.

### Code

The code for finding cell-type trees is made available in http://lcbb.epfl.ch/software.html.

## Conclusions

We studied the novel problem of inferring cell-type trees from histone modification data. We defined methods for representing the peaks as 0/1 vectors and used these vectors to infer trees. We obtained meaningful trees, conforming closely to expectations and biologically plausible, in spite of the high level of noise in the data and the very limited number of samples per cell type. Our results confirm that histone modification data contain much information about the history of cell differentiation. We carried out a number of experiments to understand the source of the noise, using replicate data where available, but also devising various noise filters. Our results show that larger replicate populations are needed to infer ancestral nodes, an important step in understanding the process of differentiation. We also discussed how cell-type trees can be used to study the evolution of cell types.

Much work remains to be done on methods for building good cell-type trees. In particular, the noisy nature of the data remains an issue. We are exploring various other data preprocessing and representation techniques which can be used for this purpose. Refining the model of gain or loss of marks may enable the use of maximum likelihood methods, which deal better with large ranges of pairwise dissimilarities and also yield more accurate inferences for internal nodes.

Since many histone marks appear independent of cell differentiation, identifying which marks are most strongly correlated with the differentiation process is of significant interest. Once such marks have been identified, reconstructing their state in ancestral nodes will enable us to identify which regions of the genome play an active role in which steps of cell differentiation.

## Methods

### Algorithm to identify interesting regions in Overlap representation

As seen before, we denote the *i*th peak in library *n* as , where  and  are the left and right endpoints (as basepair indices). We assume that we have a set of sorted peaks given to us with respect to their positions in each chromosome, otherwise we first sort the peaks.

Choose a chromosome, let *PS* be its set of peaks, set  and *z*=0, and enter the following loop:

. Set  and
2. Set  and *AS*=*AS*∪*S*.
3. If *S* is not empty, then find  and go to step 2.
4. Let  and set *PS*=*PS*−*AS*.
5. The interesting region lies between *a* and *b*, *IR*[*a*,*b*]. Let  be the data representation for *IR*[*a*,*b*] in library *n*. Set *z*=*z*+1. Set  if there is a peak in library *n* that lies in *IR*[*a*,*b*]; otherwise set  (1≤*n*≤*N*).

Repeat this procedure for all chromosomes in the genome. The above algorithm can implemented by sweeping from left to right (two ends of each chromosome) and we visit each peak only once. Therefore the algorithm takes time linear in the size of the number of all peaks in order to identify all the interesting regions. Figure S11 in Additional file 1 shows an example of interesting region as defined by overlap representation.

## Electronic supplementary material

Additional file 1: **Supplementary material.** Contains some text, Figures S1–S12, Table S1, S2. (PDF 94 KB)

Additional file 2: **H3K4me3 data: Regions of the genome (identified by Overlap representation) where ES cells (10 replicates) are all 1 and rest of the cell types (62 replicates) have all 0 (one error allowed at most on both sides).** Bed file format. (ZIP 3 KB)

Additional file 3: **H3K4me3 data: Regions of the genome (identified by Overlap representation) where ES cells (10 replicates) are all 0 and rest of the cell types (62 replicates) have all 1 (one error allowed at most on both sides).** Bed file format. (ZIP 1 KB)

Additional file 4: **H3K27me3 data: Regions of the genome (identified by Overlap representation) where ES cells (10 replicates) are all 1 and rest of the cell types (13 replicates) have all 0 (one error allowed at most on both sides).** Bed file format. (ZIP 30 KB)

Additional file 5: **H3K27me3 data: Regions of the genome (identified by Overlap representation) where ES cells (10 replicates) are all 0 and rest of the cell types (13 replicates) have all 1 (one error allowed at most on both sides).** Bed file format. (ZIP 241 bytes)

Additional file 6: **H3K4me3 data: Regions of the genome (identified by Overlap representation) where ES cells for day 0, 2, 5, 9, 14 have a pattern 01000.** Bed file format. (ZIP 10 KB)

Additional file 7: **H3K27me3 data: Regions of the genome (identified by Overlap representation) where ES cells for day 0, 2, 5, 9, 14 have a pattern 01000.** Bed file format. (ZIP 15 KB)
