# Supplementary material for: Study of cell differentiation by phylogenetic analysis using histone modification data
Source: BMC Bioinformatics. 2014 Aug 8;15(1):269. doi: 10.1186/1471-2105-15-269 (PMC4138389; doi:10.1186/1471-2105-15-269)
Supplement: Supplementary file 1 — Additional file 1: Supplementary material. Contains some text, Figures S1–S12, Table S1, S2. (PDF 94 KB) [file 12859_2014_6541_MOESM1_ESM.pdf]

Supplementary material for paper ‘Study of cell differentiation by phylogenetic analysis using histone modification data’

Nishanth Ulhas Nair, Yu Lin, Ana Manasovska, Jelena Antic, Paulina Grnarova, Avinash Das Sahu, Philipp Bucher and Bernard M.E. Moret

1 Experimental Design

For the H3K4me3, H3K27me3 ENCODE peak data we used the University of Washington (UW) ENCODE data. Both replicates used and the file type used is “narrowPeak”.
For H3K4me3 data the GEO accession numbers are: GSM945187, GSM945177, GSM945166, GSM945170, GSM945178, GSM945225, GSM945198, GSM945229, GSM945185, GSM945191, GSM945322, GSM945190, GSM945321, GSM945242, GSM945249, GSM945163, GSM945310, GSM945312, GSM945308, GSM945306, GSM945286, GSM945218, GSM945239, GSM945159, GSM945292, GSM945284, GSM945276, GSM945271, GSM945181, GSM945273, GSM945251, GSM945175, GSM945262, GSM945216, GSM945199, GSM945214, GSM945265, GSM945215.
For H3K27me3 data the GEO accession numbers are: GSM1010913, GSM945204, GSM945301, GSM945183, GSM945325, GSM945326, GSM945323, GSM945320, GSM945277, GSM945160, GSM945180, GSM945300, GSM945200.
For the H3K4me1, H3K9me3, H3K27ac ENCODE peak data we used the Broad ENCODE data. The file type used is “broadPeak”.
For H3K4me1 data the GEO accession numbers are: GSM1003535, GSM733782, GSM733705, GSM733761, GSM733661, GSM733690, GSM733710, GSM733698, GSM733649, GSM733704.
For H3K9me3 data the GEO accession numbers are: GSM1003538, GSM1003585, GSM1003485, GSM733730, GSM1003482, GSM1003517, GSM1003491, GSM1003553, GSM1003528, GSM1003531, GSM733681.
For H3K27ac data the GEO accession numbers are: GSM1003559, GSM1003459, GSM733718, GSM733660, GSM733755, GSM733666, GSM733691, GSM733763, GSM733662, GSM733674, GSM733646, GSM733739.

2 Results and discussion

Table S1: **Statistics for cell-type trees on H3K4me3 data using top peaks.** *2nd to 9th columns show the number of cells (of the same type) belonging to the largest and second-largest clades; the total number of cells of that type is in the top row. Rows correspond to various methods (OM: overlap; TP: top peaks). threshold x means that the peaks which have a (negative) log p-value $\geq x$ is used. The second last column shows the SR ratio. The last column contains the percent deviation (PD) of the distances between the leaves found using the neighbor-joining (NJ) tree from the Hamming distance between the leaves. ENCODE peak data is used. (all replicates) means all available replicates (1, 2, or 3) for each cell type is used. We can see that the method is robust to various kinds of threshold used.*

	hESC (5)	Epithelial (8)	Fibroblast (16)	Blood (2)	Astrocytes (2)	Myocytes (1)	Endothelial (2)	Skeletal Muscle (1)	*SR*	*PD* (%)
OM (all replicates)	5,0	4,2	9,4	2,0	2,0	1,0	1,1	1,0	0.78	3.88
OM (all replicates)-TP (threshold 5)	5,0	5,1	9,4	2,0	2,0	1,0	1,1	1,0	0.76	4.00
OM (all replicates)-TP (threshold 8)	5,0	6,1	9,4	2,0	2,0	1,0	1,1	1,0	0.74	3.77
OM (all replicates)-TP (threshold 10)	5,0	4,3	8,5	2,0	2,0	1,0	1,1	1,0	0.74	3.98
OM (all replicates)-TP (threshold 12)	5,0	4,2	8,5	2,0	2,0	1,0	1,1	1,0	0.73	3.85

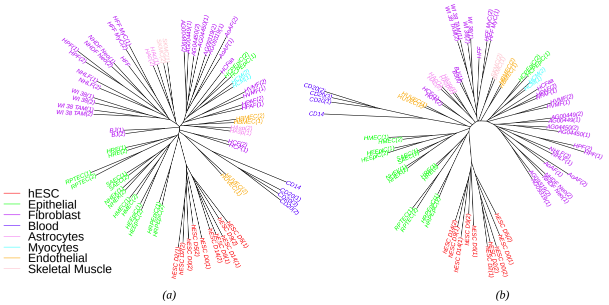

Figure S1: *Cell-type tree on H3K4me3 data (ENCODE peaks, using all replicates) on peaks with negative log p-value $\geq$ 10: (a) windowing representation, (b) overlap representation.*

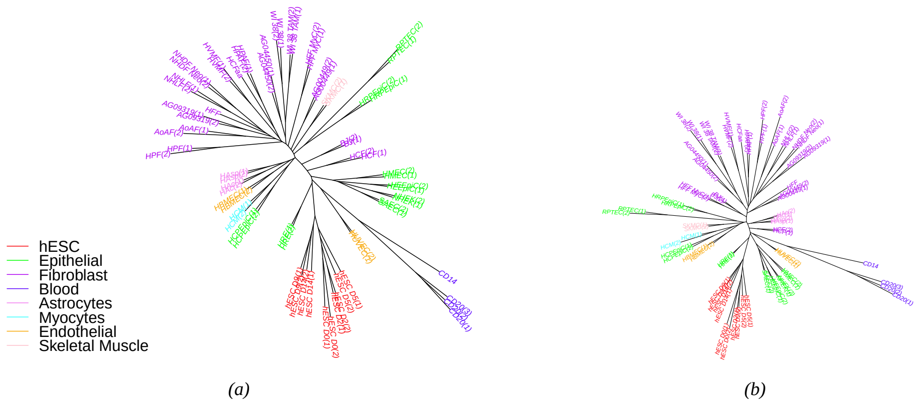

Figure S2: *Cell-type tree on H3K4me3 data (using all replicates) using (a) windowing representation (b) overlap representation. Peaks generated by MACS2 peak finder.*

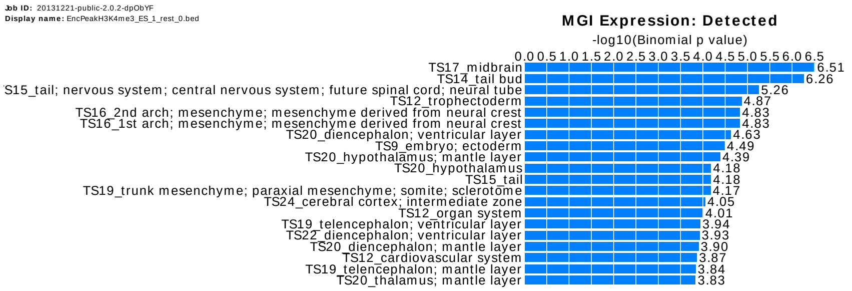

(a) MGI Expression: Detected

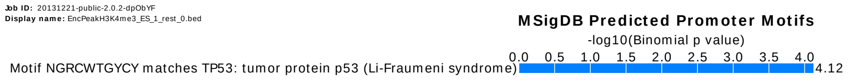

(b) MSigDB Predicted Promoter Motifs

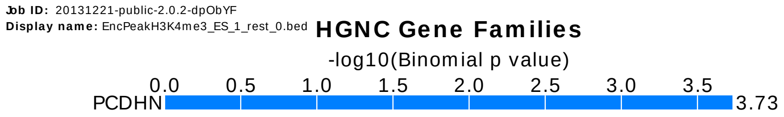

(c) HGNC Gene Families

Figure S3: *For H3K4me3 data: gene enrichment analysis containing regions of the genome where ES cells (10 replicates) are all 1 and rest of the cell types (62 replicates) have all 0 (one error allowed at most on both sides) using all replicate ENCODE peak data.*

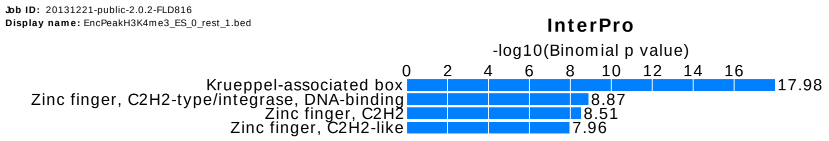

(a) InterPro

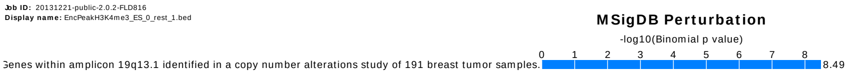

(b) MSigDB Perturbation

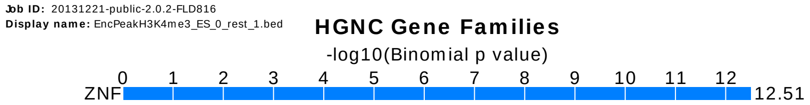

(c) HGNC Gene Families

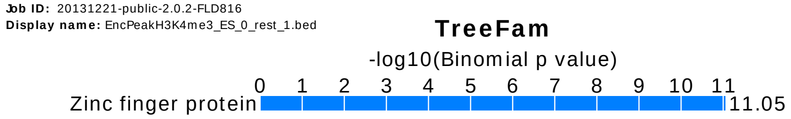

(d) TreeFam

Figure S4: *For H3K4me3 data: gene enrichment analysis containing regions of the genome where ES cells (10 replicates) are all 0 and rest of the cell types (62 replicates) have all 1 (one error allowed at most on both sides) using all replicate ENCODE peak data.*

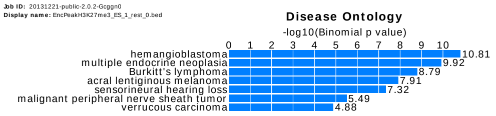

(a) Disease Ontology

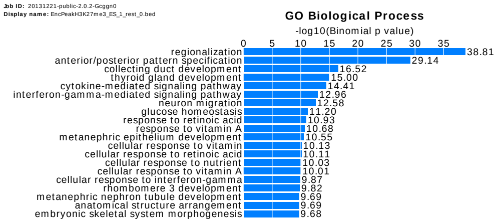

(b) GO Biological Process

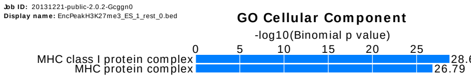

(c) GO Cellular Component

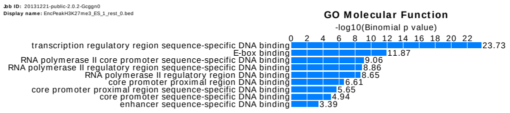

(d) GO Molecular Function

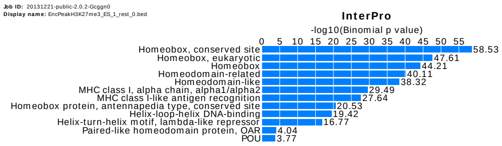

(e) InterPro

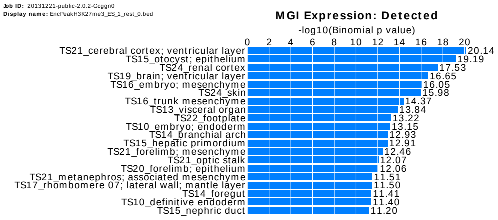

(f) MGI Expression

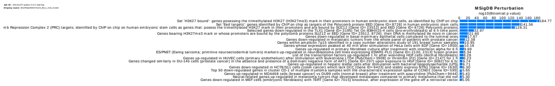

(g) MSigDB Perturbation

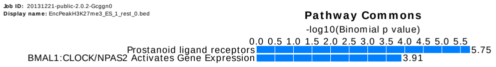

(h) Pathway Commons

Figure S5: *For H3K27me3 data: gene enrichment analysis containing regions of the genome where ES cells (10 replicates) are all 1 and rest of the cell types (13 replicates) have all 0 (one error allowed at most on both sides) using all replicate ENCODE peak data.*

Table S2: **Statistics for cell-type trees on H3K4me3 data using IDR analysis.** *2nd to 9th columns show the number of cells (of the same type) belonging to the largest and second-largest clades; the total number of cells of that type is in the top row. The second last column shows the SR ratio. The last column contains the percent deviation (PD) of the distances between the leaves found using the NJ tree from the Hamming distance between the leaves. Rows correspond to various methods OM (two replicates): overlap representation on all cell-types which have exactly two replicates (on all available peaks). OM-IDR (two replicates), (threshold x): Overlap representation used on overlapping peaks (between replicates) which have an IDR value $\leq x$ (output of IDR program). Since these are overlapping peaks the SR ratio is always 0 given the nature of overlap representation. ENCODE peak data is used. (two replicates) means all two replicates for each cell type is used (34 cell-types, 68 libraries). We considered data containing only two replicates for this work.*

	hESC (5)	Epithelial (8)	Fibroblast (14)	Blood (1)	Astrocytes (2)	Myocytes (1)	Endothelial (2)	Skeletal Muscle (1)	*SR*	*PD* (%)
OM (two replicates)	5,0	4,3	8,4	1,0	2,0	1,0	1,1	1,0	0.77	3.85
OM-IDR (two replicates), (threshold 0.01)	3,1	4,2	4,4	1,0	2,0	1,0	1,1	1,0	0	4.26
OM-IDR (two replicates), (threshold 0.1)	5,0	6,1	7,4	1,0	1,1	1,0	1,1	1,0	0	3.47
OM-IDR (two replicates), (threshold 0.25)	5,0	6,1	8,4	1,0	1,1	1,0	1,1	1,0	0	3.00

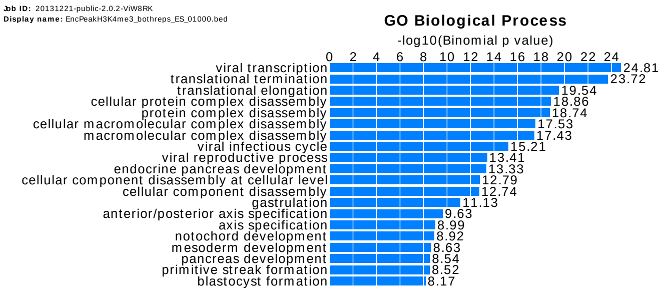

(a) GO Biological Process

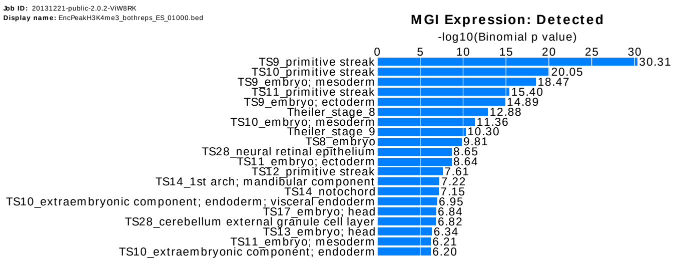

(b) MGI Expression

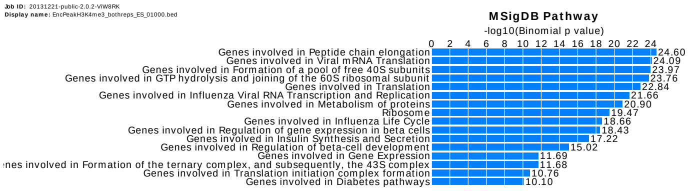

(c) MSigDB Pathway

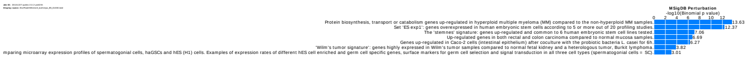

(d) MSigDB Perturbation

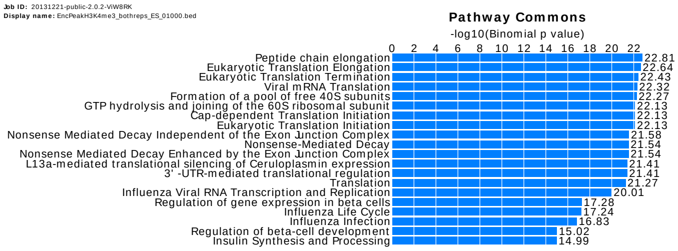

(e) Pathway Commons

Figure S6: *For H3K4me3 data: gene enrichment analysis containing regions of the genome where ES cells for day 0, 2, 5, 9, 14 have a pattern 01000. ENCODE peaks are used and only regions which have identical values of all the replicates for each cell type are considered.*

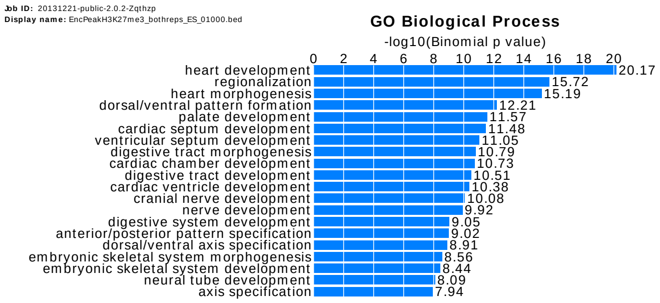

(a) GO Biological Process

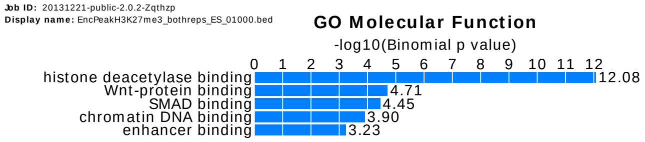

(b) GO Molecular Function

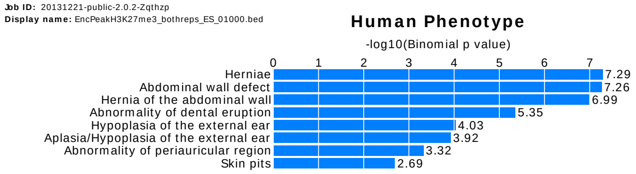

(c) Human Phenotype

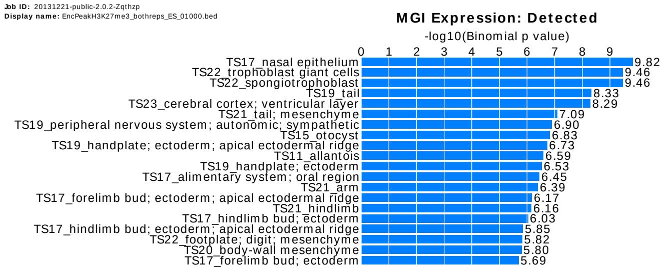

(d) MGI Expression

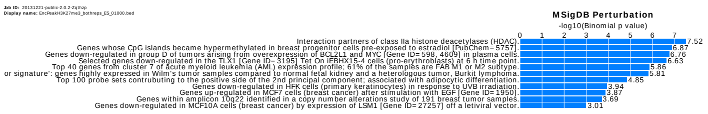

(e) MSigDB Perturbation

Figure S7: *For H3K27me3 data: gene enrichment analysis containing regions of the genome where ES cells for day 0, 2, 5, 9, 14 have a pattern 01000. ENCODE peaks are used and only regions which have identical values of all the replicates for each cell type are considered.*

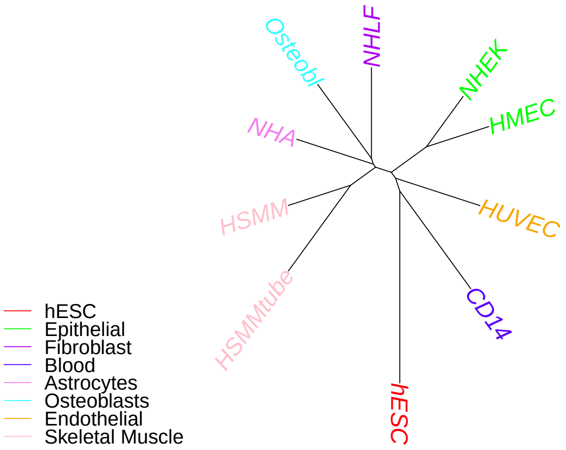

Figure S8: *Cell-type tree using windowing representation on H3K4me1 data.*

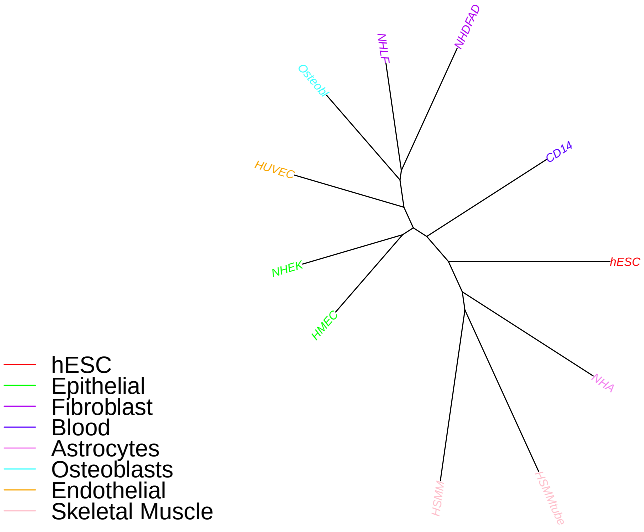

Figure S9: *Cell-type tree using windowing representation on H3K9me3 data.*

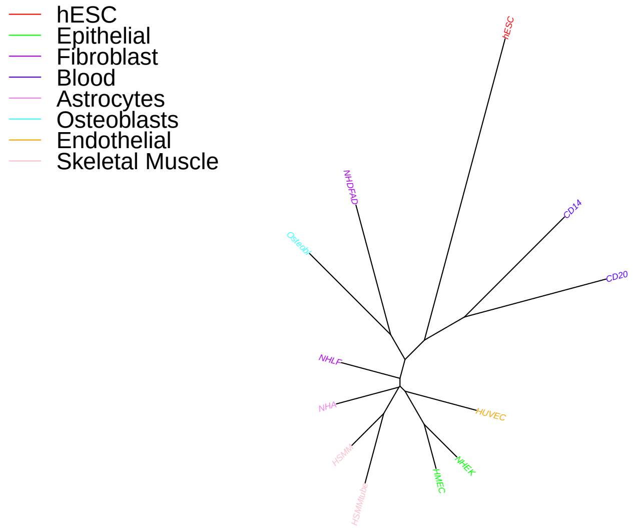

Figure S10: *Cell-type tree using windowing representation on H3K27ac data.*

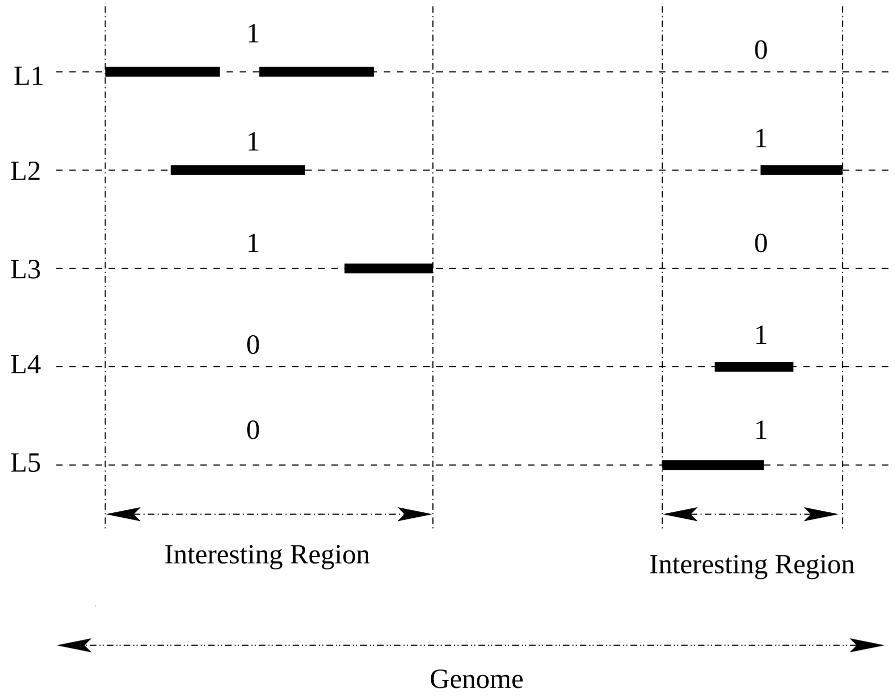

Figure S11: *An example of an interesting region as defined by overlap representation is shown. The dotted horizontal lines represents a portion of the genome. Each row stands for a separate ChIP-Seq library (L1-L5). The dark lines represent peak regions. 1 and 0 is the data representation for each library.*

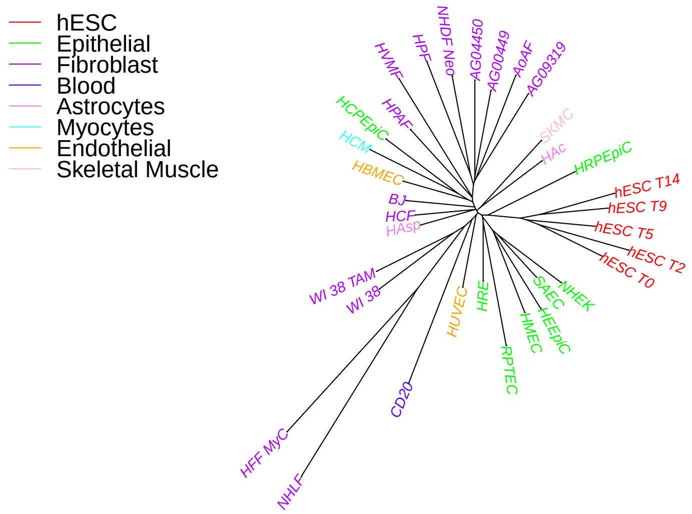

Figure S12: *Overlap representation using overlapping peaks (between replicates) which have an IDR value* ≤ 0.025 *(output of IDR program). Since the distance between replicates is 0, the two replicates of each cell type are represented in one label. ENCODE peaks used.*
